# Study of domain configurations in (Bi,Na)ZrO_3_-modified (K,Na)(Nb,Sb)O_3_ piezoelectric ceramics by acid-etching at different temperatures

**DOI:** 10.1038/s41598-020-75593-6

**Published:** 2020-10-28

**Authors:** Jialiang Zhang, Chunming Zhou

**Affiliations:** School of Physics, State Key Laboratory of Crystal Materials, Shandong University, Jinan, 250100 People’s Republic of China

**Keywords:** Materials science, Condensed-matter physics, Ferroelectrics and multiferroics

## Abstract

Domain structure often greatly affects piezoelectric performance of a ferroelectric ceramic. Accordingly, a convenient method that can well characterize the domain structure at various temperatures is highly desired for understanding the underlying mechanism. An improved acid-etching technique was recently developed for such purpose. Domain structure of poled 0.96(K_0.48_Na_0.52_)(Nb_0.96_Sb_0.04_)O_3_–0.04(Bi_0.50_Na_0.50_)ZrO_3_ ceramics with a large piezoelectric coefficient *d*_33_ of 535 pC/N was systematically investigated at three typical temperatures. It was found that domain configurations change significantly with temperature. Hierarchical nanodomain structure is widely observed in domain patterns acid-etched at 25 °C, due to the orthorhombic-tetragonal phase coexistence. By contrast, the majority part of those acid-etched at − 60 °C are simply some long parallel stripes, while a small amount of banded structure appears in broad stripes inside some grains. A nearly 63° intersectional angle is seen between two adjacent sets of parallel stripes in the domain pattern of a cuboid-shaped grain, indicating that orthorhombic phase remains down to − 60 °C. The domain patterns acid-etched at 80 °C become even simpler, mainly consisting of long parallel stripes that are several hundred nanometers wide and have quite straight edges. Fundamental issues associating with the possible domain configurations and the acid-etching were discussed on the simple mathematical basis.

## Introduction

Piezoelectric ceramics are a very important and economical type of functional materials that can develop charges under applied mechanical stress and deform under external electric field. Currently, lead zirconate titanate and their derivatives are widely used in a variety of technological products such as ultrasonic transducers, actuators and sensors, because of the performance characters of excellent piezoelectric properties and temperature stabilities. However, due to the toxicity of lead element, demands to replace them with lead-free alternatives become increasingly strong in recent years. Accordingly, (K,Na)NbO_3_-based ceramics (KNN-based ceramics) have been continuously drawing much interest as a promising type of lead-free piezoelectric materials^1^.

Domain structure often plays a significant role in affecting the piezoelectric performance of a ferroelectric ceramic. It is known that the piezoelectric properties can be generally resolved into intrinsic and extrinsic contributions^2,3^. The intrinsic part comes from relative ion shift in crystal lattice, whilst the extrinsic part arises primarily from domain-wall motion^3,4^. Particularly, the extrinsic contribution to piezoelectric coefficient *d*_33_ can take high percentages over 75% in some small-grained BaTiO_3_ ceramics^5^ and more than 70% in certain Pb(Zr,Ti)O_3_-based ceramics^6^.

How to visualize thereby characterizing the domain structure, particularly catching the subtle changes in it with temperature, is believed to be significantly important for the understanding of piezoelectric performance. Morphological observation of chemically etched surface with an optical microscope or a scanning electron microscope (SEM), structural observation of thin specimens prepared by mechanical polishing and ion milling with a transmission electron microscope (TEM) and detection of surface polarization distribution with a piezoelectric force microscope (PFM), are presently the three most commonly used methods for the visualization of domain structure. Each of these observation methods has its own advantages and shortcomings. Surface chemical etching bases on the different etching rates of the opposite polarities (the positive end and the negative end) of a polarizations dipole^7–10^. Aqueous solutions of HF, HCl and HNO_3_ acids are often used as the etchant. The acid-etching method allows easily observing the domain patterns of a large number of grains thereby drawing a reliable conclusion on a basis of statistical analysis^5^. The disadvantages are that it usually cannot provide the information about the polarization orientations in the domains. The TEM observation has a very high resolution but requires very thin specimens. It is quite sensitive to the specimen preparation, during which domain structure may change irreversibly due to the stresses of mechanical polishing and focused ion-beam milling. The PFM observation is also one of the most useful methods, because of the simple specimen preparation and usually no need to operate in vacuum. It allows not only the visualization of domain structure, but also the quantitative determination of the polarization orientation by simultaneous analysis of out-of-plane and in-plane piezoresponse signals^9–11^. However, limited view window and quite time consuming seem to be the disadvantages.

Numerous studies on domain structure have been performed for the various compositional KNN-based ceramics so far, mostly by TEM^12–18^, PFM^11,19–23^ or the combination of TEM and PFM^24–29^. A few of these studies investigated the evolution of domain configurations at room temperature and above it^13–15,21,23,25,26^. The attempt of acid-etching was made quite earlier for a pure KNN ceramic^30^. However, most of these previous studies were conducted for unpoled KNN-based ceramics. More efforts should be paid to the domain structure of poled KNN-based ceramics, as it is known that ferroelectric ceramics show the piezoelectricity only after poling while domain configurations may change greatly upon poling. On the other hand, the acid-etching observation method is considered as a relatively reliable technique for revealing the true domain structure of a ferroelectric ceramic^31^. Polycrystalline grains inside the ceramic bulk are clamped three-dimensionally by their neighboring grains, whereas those at the surface experience only a half clamping from one side. Due to this clamping difference, domain structure in the grains at the surface may differ significantly from that in the inner grains. The situation may become even more severe for the case of ceramic lamellas used in the TEM observation, as the thickness is usually less than the grain sizes. The grains in these ceramic lamellas are free of clamping in the thickness direction and thereby being clamped in two dimensions only. This may lead to a substantial change in domain structure even if no additional stress is introduced during the specimen preparation, where mechanical polishing and ion milling are adopted. It is obvious that the reliability of a result is greatly dependent on the character of ceramic specimens used in the study of domain structure. Ideally, the specimen preparation that does not alter the frozen-in state is required so that the observed domain patterns can represent the frozen-in domain configurations of grains inside the ceramic^31^. For the reasons just described, the author’s group paid continuously their study efforts onto the domain structure of poled KNN-based ceramics by means of the acid-etching in the past few years^32–36^.

It is noteworthy that no studies had been performed for the domain structure of (K,Na)NbO_3_-based ceramics at low temperatures due to the lack of a proper technological way. Apparently, this is not a satisfactory situation for the comprehensive understanding about the underlying physical mechanisms of piezoelectric performance. Therefore, a feasible method that can well characterize the domain structure at different temperatures is highly needed. To meet the requirement, we recently made a preliminary attempt in our previous study to extend the acid-etching technique such that it can be used at various temperatures^37^. In this study, we further optimized this technique and pursued a more systematical study on domain configurations of poled 0.96(K_0.48_Na_0.52_)(Nb_0.96_Sb_0.04_)O_3_–0.04(Bi_0.50_Na_0.50_)ZrO_3_ ceramics (which possess a significantly large piezoelectric coefficient *d*_33_ of 535 pC/N) at − 60 °C, 25 °C and 80 °C, respectively. The results showed that the newly developed method is not only effective but also reliable for conveniently investigating the domain structure at different temperatures. In addition, fundamental issues relevant to the domain structure and the acid etching were discussed on the simple mathematic basis.

## Results and discussion

**Figure 1:**
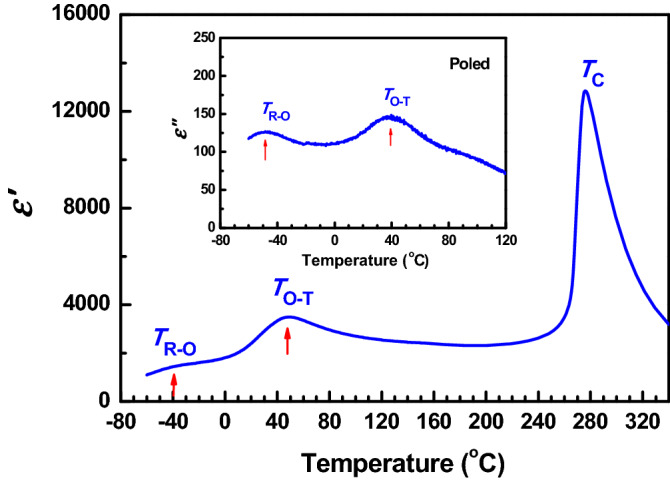
The *ε′* vs. temperature dependence of poled KNNS-BNZ ceramic, which was measured upon heating. The inset shows the curve of *ε′′* vs. temperature dependence.

The KNNS-BNZ ceramics used in this study show a quite large *d*_33_ value of 535 pC/N at room temperature and a mass density *ρ* of 4.548 g/cm^3^, which corresponds to a relative density *ρ′* of approximately 98.7%^36^. Therefore, these KNNS-BNZ ceramics might be taken as a good representative of those high-*d*_33_ KNN-based piezoelectric ceramics obtained in recent years. Figure 1 show the curves of real and imaginary parts of relative dielectric permittivity (*ε′* and *ε′′,* respectively) versus temperature dependences measured for the poled KNNS-BNZ ceramic at 100 kHz upon heating under a rate of 1.0 °C/min. Two peaks are seen in these curves in the temperature range below 120 °C, which correspond to the rhombohedral-orthorhombic (R-O) phase transition and orthorhombic-tetragonal (O-T) phase transition, respectively. The corresponding peak temperatures, *T*_R-O_ and *T*_O-T_, are about − 40 °C and 49 °C in the ε′-temperature curve, and are approximately − 47 °C and 39 °C in the *ε′′*-temperature curve, respectively. Compared to those prepared under shorter sintering time^35^, the present KNNS-BNZ ceramics shows a slightly lower *T*_O-T_ but a little larger *ρ′* and a further enhanced *d*_33_. The X-ray diffraction (XRD) analysis indicated that poled KNNS-BNZ ceramics at room temperature are in O–T phase coexistence with the phase volume ratios of *V*_O_ = 54.2% and *V*_T_ = 45.8%, respectively^36^.

The evolution of domain structure with temperature in poled KNNS-BNZ ceramics was explored in this study through examining the domain patterns that are revealed by acid-etching at room temperature, − 60 °C and 80 °C, respectively. These three temperatures were chosen by taking both the character of phase transitions of the KNNS-BNZ ceramics and the experimental feasibility into consideration. Poled KNNS-BNZ ceramics are in the O–T phase coexistence state of slightly more orthorhombic phase at 25 °C according to the XRD analysis, and are expected from the dielectric measurement data to be mainly of rhombohedral phase at − 60 °C and tetragonal phase at 80 °C, respectively. The low etching temperature was set to − 60 °C so that the possible freezing of the acid aqueous solution can be avoided and the experiment temperature is in the operational temperature limit of the Espec SU-261 chamber. The high etching temperature was set to 80 °C from safety consideration that the danger of highly corrosive acid due to the possible evaporation might occur at further high temperatures. In addition, the optimum etching time at 80 °C was found to be about 12 s, which is quite short but still in the experimentally convenient range of precise control and good repeatability. In contrast, the optimum etching time was confirmed to be about 46 min at − 60 °C and about 4 min at 25 °C. Thus, the acid-etching rate changes greatly with temperature.

**Figure 2:**
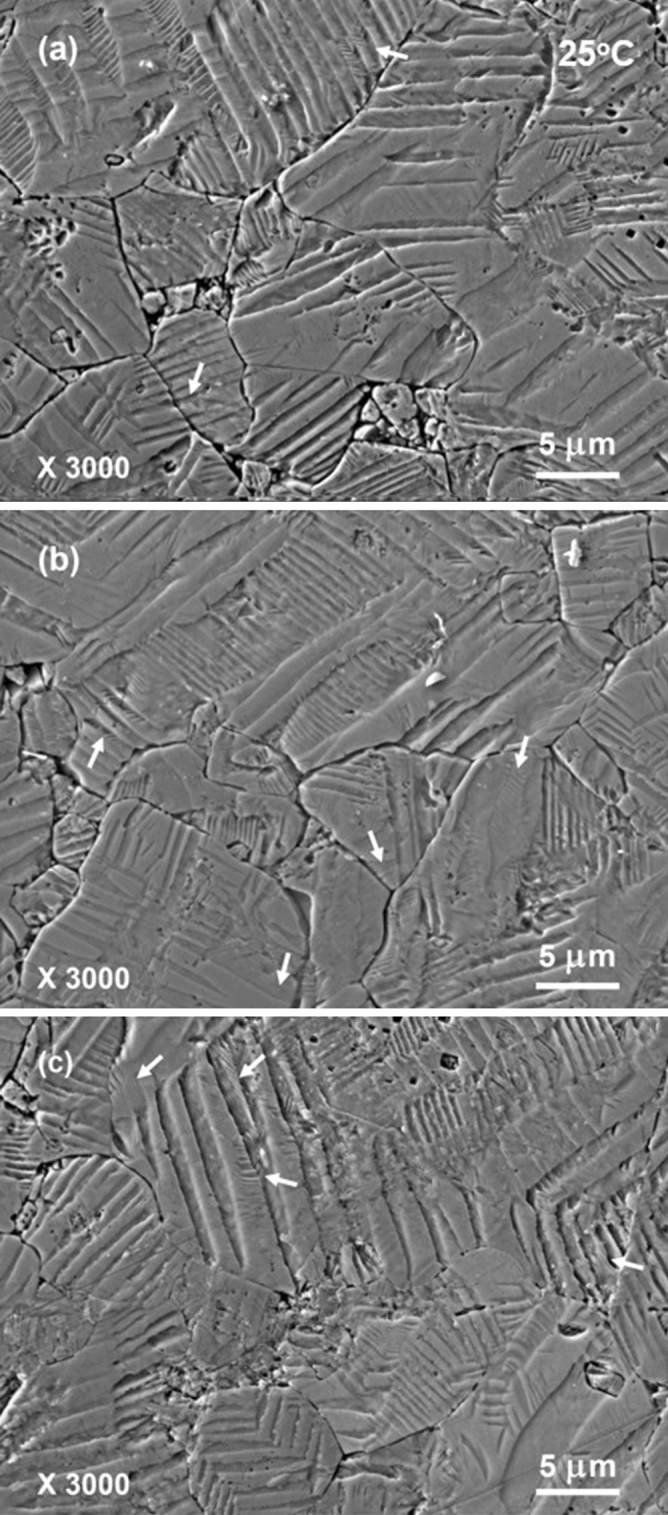
Typical SEM images of domain patterns observed in a poled KNNS-BNZ ceramic specimen that was acid-etched at room temperature. The arrows show the locations of hierarchical nanodomain structure.

**Figure 3:**
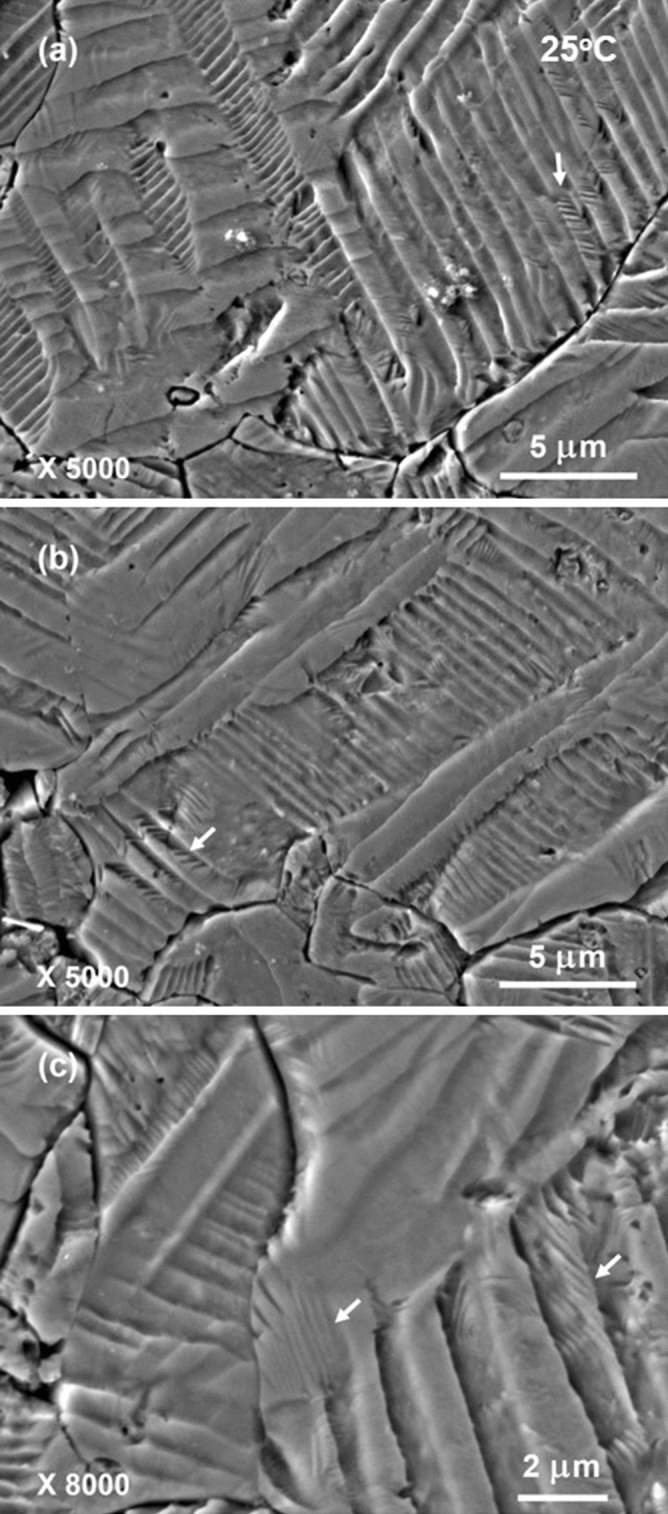
High-magnification SEM images of domain patterns that were acid-etched at room temperature. The images were observed in the same specimen shown in Fig. 2.

Figures 2 and 3 show some typical SEM images of domain patterns that were acid-etched at room temperature. Grain boundaries and microstructure could be clearly seen also in these images. The grains in microstructure show typically large sizes of about 10 μm. Long stripes with quite broad widths are observed almost in every individual grain. These stripes are several hundred nanometers to several micrometers wide and traverse the large part or even the whole of a grain. For description convenience, we call such type of long and broad stripes as “bands”^35^. Frequently, fine parallel strips are further recognized inside some of these bands. Most of fine parallel strips are in the shapes of short segments and intersect with the side edges of corresponding matrix bands. Part of fine parallel strips have their widths even narrower than 100 nm. Such type of domain configurations that fine and short strips exist in broad stripes correspond to the hierarchical nanodomain structure reported earlier in literature and is believed to play an important role in enhancing the piezoelectric properties^12,14,15,26,34–39^. As shown in Fig. 3c, a small volume fraction of fine parallel strips that have quite long lengths and are aligned nearly parallel to the band edges can also be occasionally seen. The physical origin for the formation of hierarchical nanodomain structure is considered to arise from the change in polarization anisotropy^38^. Low polarization anisotropy in the phase coexistence region results in a decrease of domain wall energy and a consequent reduction of domain widths. It is worth noting that part of band edges are in curved shapes, perhaps due to the low polarization anisotropy associating with the O–T phase coexistence at room temperature. Interestingly, irregularly shaped watermarks that correspond to 180°-domains are frequently observed in unpoled ferroelectric ceramics^30,34,35,39^ are hardly observed in the present domain patterns, indicating that the poling of KNNS-BNZ ceramics is complete. Furthermore, it is worth noting that there are also a large number of bands inside which no fine nanodomain structure can be found completely or partly. Possible mechanism resulting in this phenomenon will be discussed later.

**Figure 4:**
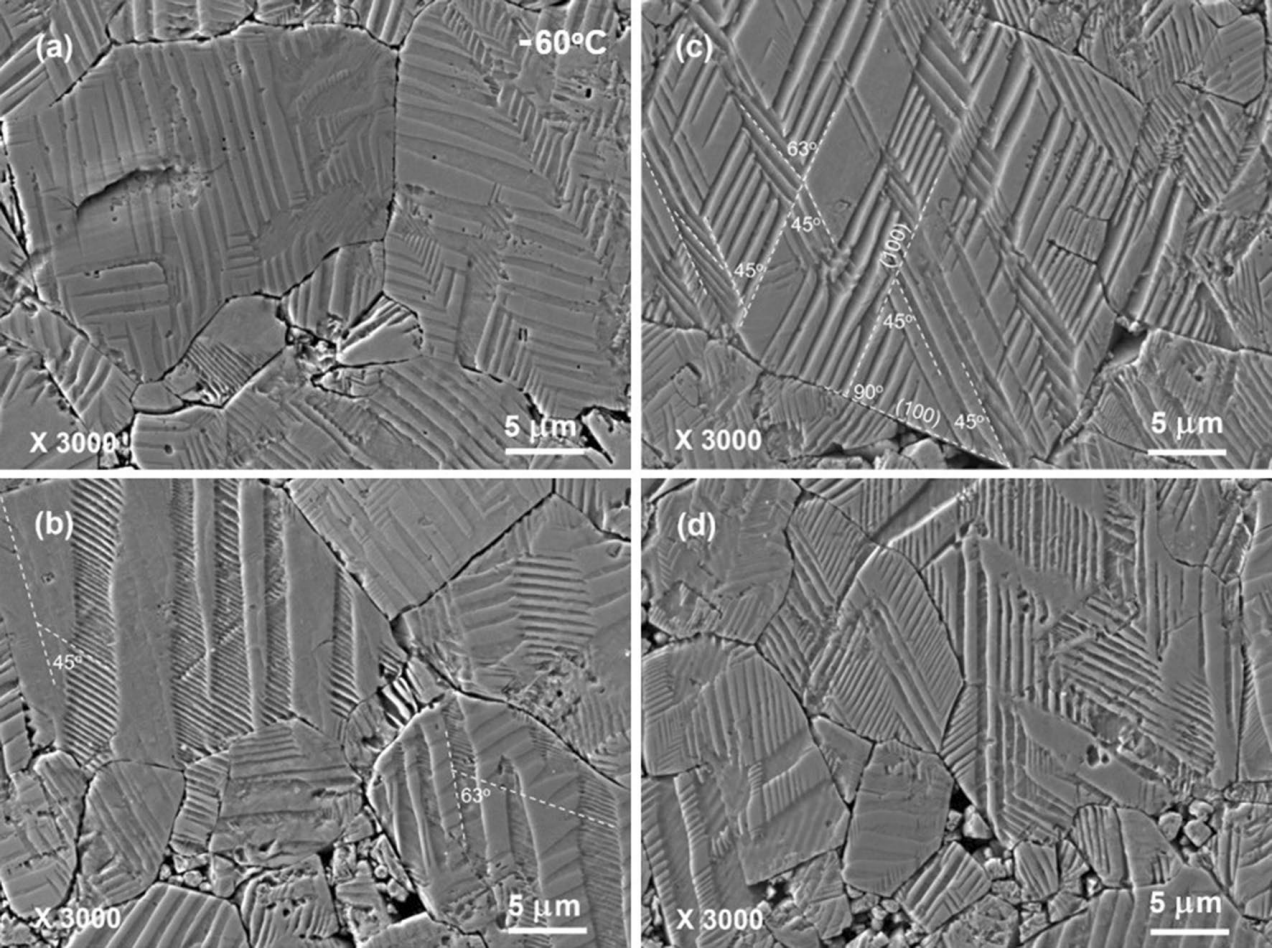
Typical SEM images of domain patterns that were acid-etched at − 60 °C. Dashed lines in panels (**b**) and (**c**) show the plane directions of domain walls. A regular grain with the cuboidal shape is seen in (**c**).

**Figure 5:**
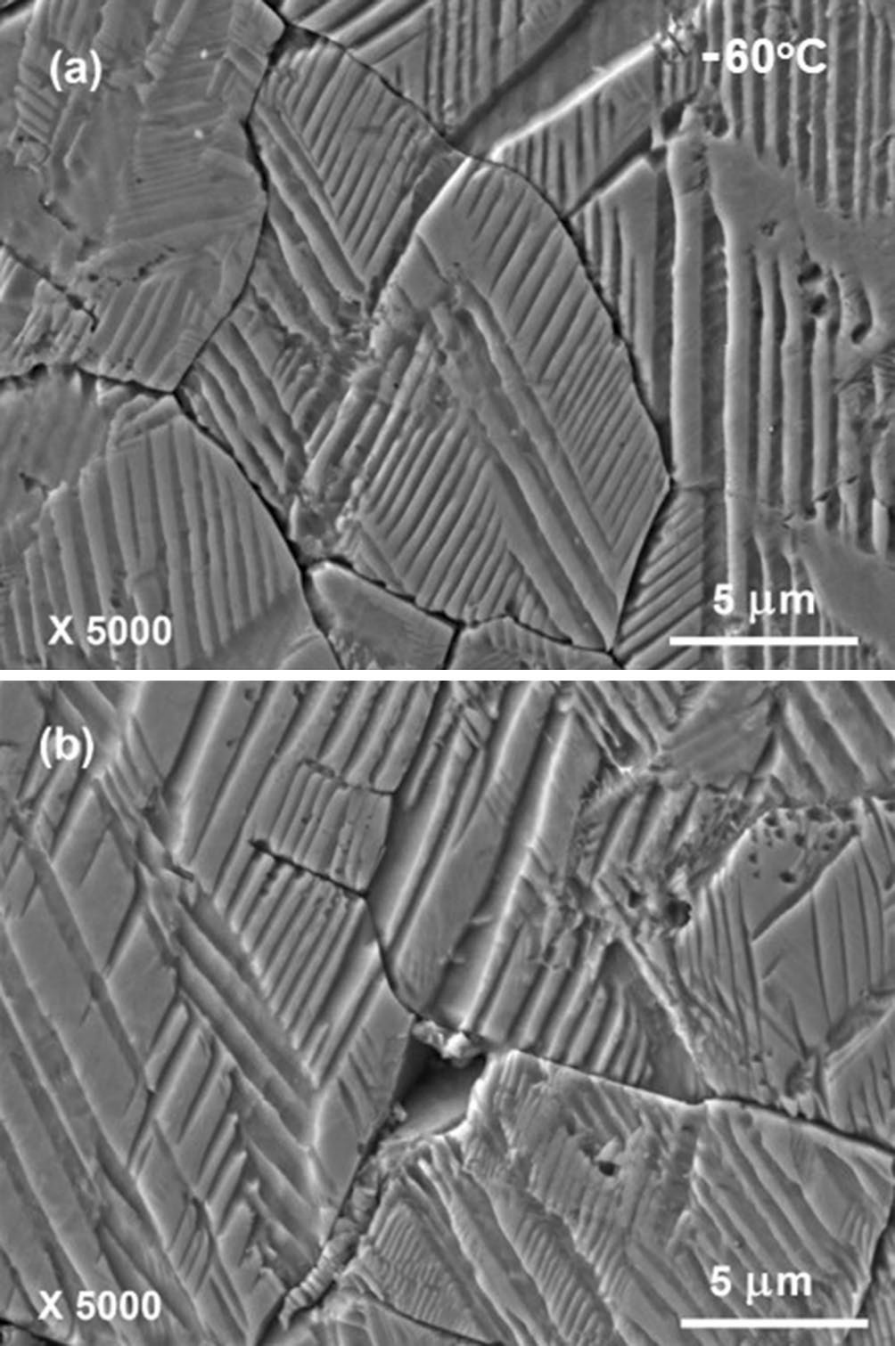
High-magnification SEM images of domain patterns that were acid-etched at − 60 °C. The images were observed in the same specimen shown in Fig. 4.

In contrast to those complicated domain patterns that were acid-etched at room temperature, the domain patterns acid-etched at − 60 °C are much simpler, as shown in Figs. 4 and 5. Long but simple parallel stripes are more often seen in the domain patterns. Most of these stripes are several hundred nanometers in width. Banded structure with short parallel stripes appearing in broad stripes of several micrometers wide can be also found in some grains, as demonstrated in Fig. 4b. The short parallel stripes within the bands are several hundred nanometers on average. Nevertheless, fine hierarchical structure with domains width less than one hundred nanometers is hardly observed. As shown in Fig. 4b and 4c, the angles between the adjacent sets of parallel stripes in some regularly cuboid-shaped grains are approximately 45° or 63°. It indicates that orthorhombic phase in poled KNNS-BNZ ceramics remains at least partly down to − 60 °C. This seems to be against the speculation made from the result of dielectric measurement where the *T*_R-O_ values determined upon a heating process are higher than − 60 °C, but largely resembles the above described case of *T*_O-T_. Besides, as shown by the domain patterns in Fig. 4, part of the band edges inside some grains are not strictly straight lines but curved ones, reflecting the character of R–O phase coexistence at − 60 °C.

**Figure 6:**
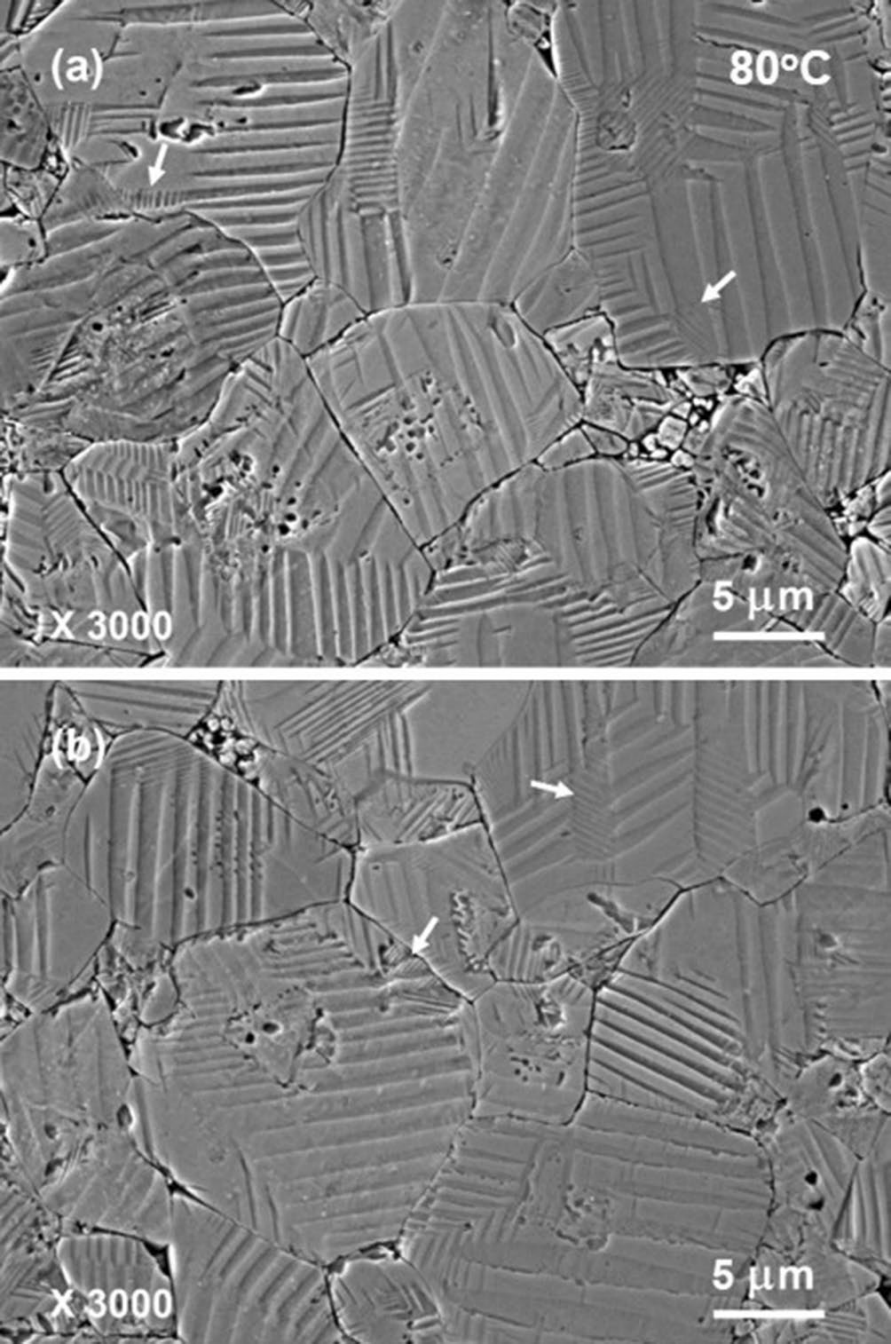
Typical SEM images of domain patterns that were acid-etched at 80 °C. The arrows show the locations of hierarchical domain structure.

**Figure 7:**
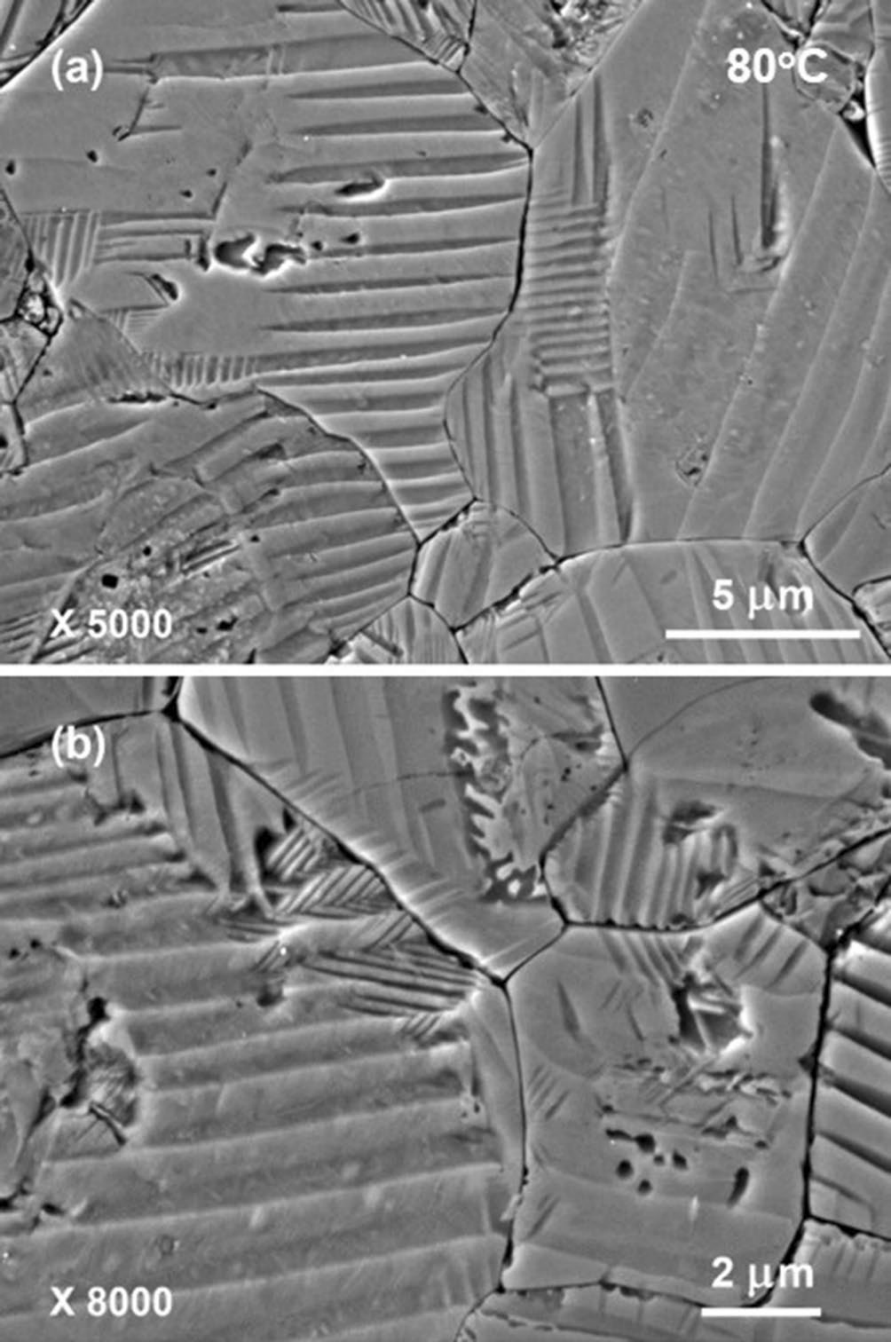
High-magnification SEM images of domain patterns that were acid-etched at 80 °C. The images were observed in the same specimen shown in Fig. 6.

Figures 6 and 7 present some typical SEM images of domain patterns that were acid-etched at 80 °C. Domain patterns become coarse and even simpler, in comparison to those shown in Fig. 2. Simple patterns consisting of long stripes are observed in most of the grains. The long stripes have a rather uniform width distribution, while majority of the stripes are several hundred nanometers wide. Complex hierarchical domain structure that short parallel segments appear inside the broad stripes is only occasionally seen in small parts of some grains. Besides, the edges of the stripes become relatively straight. It possibly reflects the fact that the majority part of a poled KNNS-BNZ ceramic has transformed into tetragonal phase at 80 °C, which is consistent with that expected from the result of dielectric measurement. In addition, watermarks are still not recognized in the domain patterns, indicating that no depoling has occurred and polarization of poled KNNS-BNZ ceramics keep stable at least up to 80 °C.

In order to get a better understanding about the various domain patterns obtained at different temperatures, several fundamental issues concerning domain structure and acid etching will be discussed below. The characterization of domain structure with acid-etching technique is based on the different etching rates between the positive and negative ends of ferroelectric dipoles at the polished surface (the observation plane). Domains that are actually of lamellar shape in three-dimensional space^39^ are observed as alternating light and dark parallel stripes in the domain patterns. It is known that there are 8, 12 and 6 energetically equivalent orientations of spontaneous polarizations in each single phase of rhombohedral, orthorhombic and tetragonal phases, respectively. In the framework of pseudocubic denotation, the equivalent orientations of spontaneous polarizations are along the <111> *c* directions for rhombohedral phase, along <110> *c* for orthorhombic phase and along <100> *c* for tetragonal phase, respectively. Three types of domain walls (the 70.5°, 109.5° and 180° types, respectively) are permitted in rhombohedral phase. Four types of domain walls (the 60°, 90°, 120° and 180° types, respectively) are allowed in orthorhombic phase, while only two types of domain walls (the 90° and 180° types) are permissible in orthorhombic phase.

**Figure 8:**
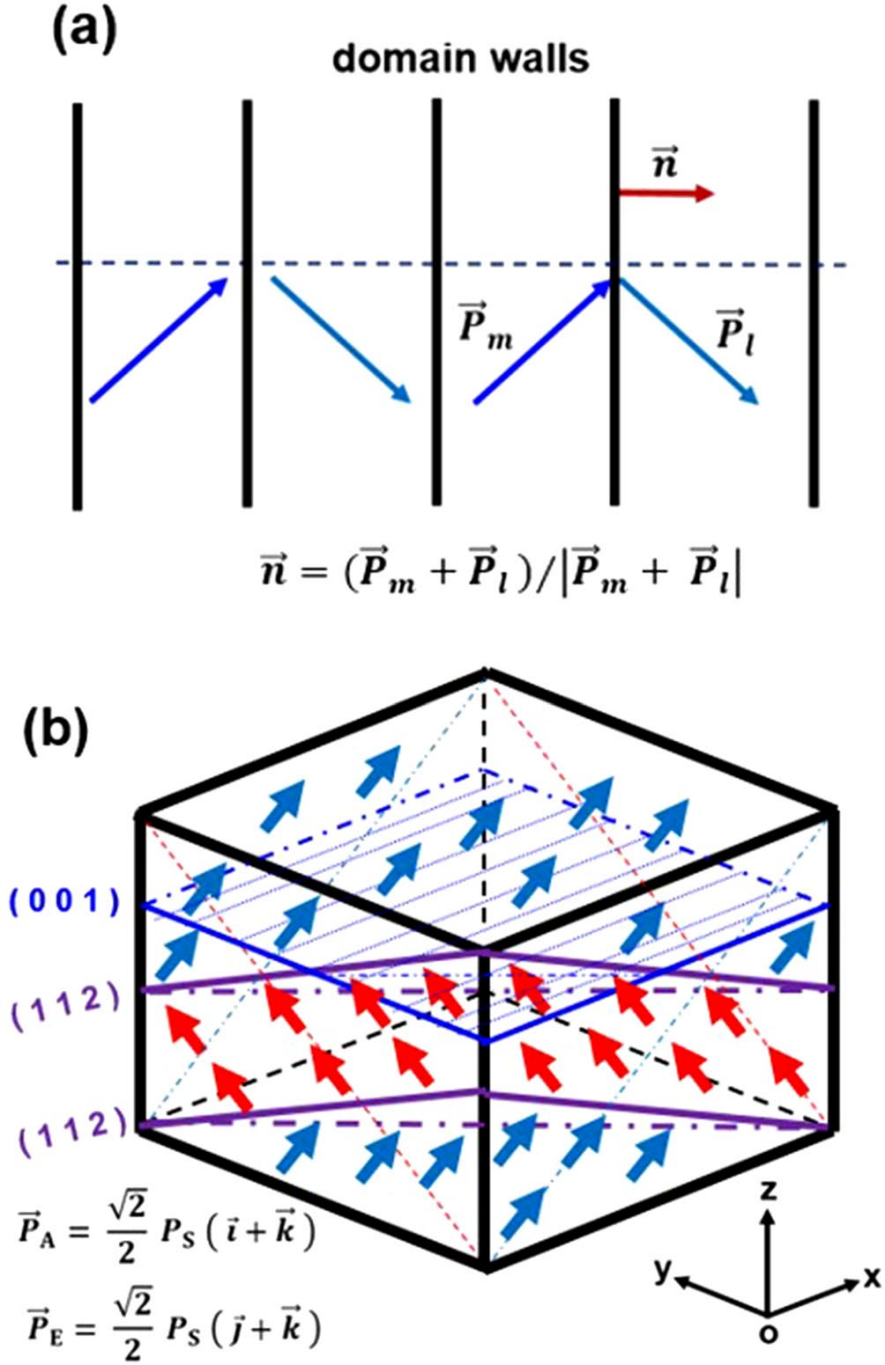
Schematic diagrams. (**a**) A head-to-tail arrangement of spontaneous polarizations in adjacent domains and the corresponding domain walls. (**b**) An example, showing that domain walls may not be recognized by acid-etching in some observation planes.

Because the uncharged domain walls are believed to be energetically more stable than those charged domain walls^31^, our discussion will be limited only to the cases of uncharged domain walls below. A head-to-tail arrangement and equal projections to the normal direction are essential conditions for two adjacent polarizations of ${\overrightarrow{{\varvec{P}}}}_{{\varvec{m}}}$ and ${\overrightarrow{{\varvec{P}}}}_{{\varvec{l}}}$ forming an uncharged non-180° domain wall, as shown in Fig. 8a. Thus, the normal direction of domain wall for such configuration should be

1 $$\overrightarrow{{\varvec{n}}}=({\overrightarrow{{\varvec{P}}}}_{{\varvec{m}}}+{\overrightarrow{{\varvec{P}}}}_{{\varvec{l}}})/\left|{\overrightarrow{{\varvec{P}}}}_{{\varvec{m}}}+\boldsymbol{ }{\overrightarrow{{\varvec{P}}}}_{{\varvec{l}}}\right|.$$

The intersection angle ϕ between the two sets of parallel planes (domain walls) with their normals $\overrightarrow{{\varvec{r}}}$ and $\overrightarrow{{\varvec{s}}}$ is calculated as

2 $$\mathbf{cos}{\varvec{\phi}}=\boldsymbol{ }\overrightarrow{{\varvec{r}}}\bullet \overrightarrow{{\varvec{s}}}/\left|\overrightarrow{{\varvec{r}}}\right|\bullet \left|\overrightarrow{{\varvec{s}}}\right|.$$

This is the intrinsic angle observed in a plane that is perpendicular to the two set of parallel planar domain walls. However, if the observation plane is not perpendicular to the set of parallel planar domain walls, the observed intersection angle should differ from ϕ. In general, in a third arbitrary plane with its normal direction of $\overrightarrow{{\varvec{t}}}$, an angle $\mathsf{\varphi }$ is observed instead of ϕ. It can be calculated by:

3 $$\mathbf{cos}\mathsf{\varphi }={\overrightarrow{{\varvec{u}}}}_{{\varvec{r}}{\varvec{t}}}\bullet {\overrightarrow{{\varvec{u}}}}_{{\varvec{s}}{\varvec{t}}}/\left|{\overrightarrow{{\varvec{u}}}}_{{\varvec{r}}{\varvec{t}}}\right|\bullet \left|{\overrightarrow{{\varvec{u}}}}_{{\varvec{s}}{\varvec{t}}}\right|.$$

where ${\overrightarrow{{\varvec{u}}}}_{{\varvec{r}}{\varvec{t}}}$ and ${\overrightarrow{{\varvec{u}}}}_{{\varvec{s}}{\varvec{t}}}$ are:

4 $$\vec{\user2{u}}_{{{\varvec{rt}}}} = \user2{ \vec{r}} \times \user2{\vec{t}, \vec{u}}_{{{\varvec{st}}}} = \user2{ \vec{s}} \times \vec{\user2{t}}.$$

Possible values of intrinsic angle ϕ are calculated to be approximately 45° and 90° in rhombohedral phase, be approximately 35.3°, 45°, 65.9° and 90° in orthorhombic phase, and be approximately 45° and 90° in tetragonal phase, respectively. Accordingly, possible $\mathsf{\varphi }$ values observed in $\left\{0 0 1\right\}$ planes are limited to 45° and 90° in rhombohedral phase, 26.6°, 45°, 63.4° and 90° in orthorhombic phase, and 45° and 90° in tetragonal phase, respectively.

Since the crystallographic orientations are randomly aligned in a non-textured ceramic, the crystallographic planes exposed to the observation plane are diverse for different polycrystalline grains. Usually, it is difficult to assign the polarization directions of domains without knowing the specific crystallographic axes for a polycrystalline grain. This seems to be a shortcoming for the acid-etching observation method in comparison to the AFM and TEM observation ones. Occasionally, however, when some grains with regular shape in microstructure are seen in the observation plane, valuable information could be drawn. As shown in Fig. 4c, a rectangular grain is observed in microstructure. The grain boundaries and the etched surface of this grain are judged to correspond to the $\left\{0 0 1\right\}$ planes, as these planes are often the crystal growth faces for a perovskite-structured oxide. Part of stripes are either parallel with or perpendicular to its grain boundaries. Further, the intersection angels of parallel stripes with grain boundaries and the intersection angles between adjacent sets of parallel stripes inside the grain show some fixed values of approximately 45°, 63° and 90°, respectively. Therefore, it is judged that this grain is in orthorhombic phase.

On the other hand, whether two adjacent domains of polarizations ${\overrightarrow{{\varvec{P}}}}_{{\varvec{m}}}$ and ${\overrightarrow{{\varvec{P}}}}_{{\varvec{l}}}$ can be discerned by acid etching totally depends on the difference of surface charge density on the observation plane due to the two polarizations. The difference of surface charge density at the two sides of the domain wall on an observation plane with its normal direction $\overrightarrow{{\varvec{t}}}$ can be calculated by.

5 $$\Delta {\varvec{\sigma}}_{{\varvec{t}}} = \vec{\user2{P}}_{{\varvec{m}}} \cdot \vec{\user2{t}} - \vec{\user2{P}}_{{\varvec{l}}} \cdot \vec{\user2{t}}.$$

The larger the absolute value of ${\Delta{\varvec{\sigma}}}_{{\varvec{t}}}$ is, the clearer the etched domain pattern should be. Conversely, if ${\Delta{\varvec{\sigma}}}_{{\varvec{t}}}$= 0, then two domains cannot be discerned in that observation plane by the acid-etching. The phenomenon that fine hierarchical structure is not observed for part of bands in domain patterns shown in Figs. 2 and 3 seems to be explainable from this aspect. To better understanding this, a schematic diagram presented in Fig. 8b gives a specific example. Now, consider the case that adjacent domains with polarizations of ${\overrightarrow{{\varvec{P}}}}_{\mathbf{A}}=\boldsymbol{ }\frac{\sqrt{2}}{2}\boldsymbol{ }{{\varvec{P}}}_{\mathbf{S}}\boldsymbol{ }\left(\boldsymbol{ }\widehat{{\varvec{i}}}+\widehat{{\varvec{k}}}\right)$ and ${\overrightarrow{{\varvec{P}}}}_{{\varvec{E}}}=\boldsymbol{ }\frac{\sqrt{2}}{2}\boldsymbol{ }{{\varvec{P}}}_{\mathbf{S}}\boldsymbol{ }\left(\boldsymbol{ }\widehat{{\varvec{j}}}+\widehat{{\varvec{k}}}\right)$ form the domain walls parallel to the $\left(1 1 2\right)$ plane in orthorhombic phase. Then, etched pattern of parallel domain stripes will be distinguished in observation planes that are parallel to the $\left(1 0 0\right)$ and $\left(0 1 0\right)$ planes but not be unrecognizable in the observation plane that is parallel to the $\left(0 0 1\right)$ and $\left(1 1 1\right)$ planes.

## Conclusions

An improved acid-etching technique that is convenient and reliable for characterizing the domain structure at different temperatures was successfully developed in our recent study. Examination on evolution of domain configurations in poled KNNS-BNZ ceramics was performed by comparing the various domain patterns that are acid-etched at − 60 °C, 25 °C and 80 °C, respectively. The study showed that both acid-etching rate and domain configurations change significantly with temperature. Hierarchical nanodomain structure is widely observed in part of broad stripes at 25 °C, which should be the consequence of orthorhombic-tetragonal phase coexistence. In contrast to those complicated domain patterns observed at 25 °C, the acid-etched domain patterns at − 60 °C become much simpler. The latter domain patterns usually consist of simple and long parallel stripes, while only a small portion of banded structure appear in broad stripes in some grains. An intersectional angle of nearly 63° was observed between adjacent sets of parallel stripes in the domain patterns of cuboid-shaped grains, which indicates that the poled KNNS-BNZ ceramics at − 60 °C are still at least partly in orthorhombic phase. The domain patterns acid-etched at 80 °C are typically composed of parallel long stripes, mostly being several hundred nanometers wide and showing quite straighter edges. Hierarchical domain structure is only occasionally observed at this temperature. In general, the obtained results support the previous speculation that the piezoelectric properties and the piezoelectric temperature stabilities of KNNS-BNZ ceramics are greatly affected by the phase transitions and the corresponding domain structure. Some fundamental issues that are related to the domain configurations and the acid-etching were treated on the simple mathematical basis.

## Methods

The 0.96(K_0.48_Na_0.52_)(Nb_0.96_Sb_0.04_)O_3_–0.04(Bi_0.50_Na_0.50_)ZrO_3_ (hereinafter denoted as KNNS-BNZ) ceramics were prepared via the conventional solid-state reaction route as per previous studies^36^. Starting raw materials of reagent-pure carbonate and oxide powders were precisely weighed in desired stoichiometric ratio and ball-milled in nylon jars with alcohol as medium. After drying, the obtained mixture was calcined at 890 °C for 8 h. Followed was a second round of ball milling and drying. The resultant ceramic powder was then granulated and pressed at 300 MPa into small thin disks. Lastly, the small thin disks were sintered by a two-step temperature profile in ambience. In this sintering process, the temperature was first quickly raised 1170 °C, kept for 5 min, then cooled down in 5 min to 1070 °C and maintained for 20 h before subsequent natural cooling.

The KNNS-BNZ ceramic specimens with fired silver-paste electrodes were poled under a direct current electric field of 4 kV/mm for 30 min in silicone oil at 25 °C. Piezoelectric coefficient *d*_33_ was evaluated by a YE2730A Berlincourt-type *d*_33_-meter. Dielectric temperature dependence was measured with an Agilent 4294A precision impedance analyzer.

Domain structure was investigated by an improved acid-etching technique at different temperatures. The necessary preparing work before the acid-etching, such as lapping, polishing and untrasonic washing, was performed at room temperature. Electrodes and surface layers of poled ceramic specimens were first lapped off with emery abrasive papers. Surface polishing was then implemented by suspension liquid of fine silica powder on polyurethane cushions. Acid-etching was performed immediately after the lapping and polishing. The time duration from the start of electrode lapping to the beginning of acid etching was quite short in our study. The temperature-controlled acid-etching procedure was carried out with the aid of an Espec SU-261 chamber. A polyethylene container that has a cap to avoid any possible leaking of acid vapor at high temperatures and a specially designed gadget facilitating the experimental specimens to be easily put into and be picked out from the acid aqueous solution was used in this acid-etching procedure. In practical experiments, the polyethylene container was first partially filled by a mixed solution of aqueous HCl acid and aqueous HF acid (which have the mass concentrations of about 37% and 40%, respectively) in the volume ratio of 1:1. The polished ceramic specimens and the polyethylene container filled with the mixed acid aqueous solution were together put into the temperature-controlled chamber. After the temperature got stabilized, the specimens were dipped into the mixed acid solution and etched in it for desired time lengths. Subsequently, the acid-etched specimens were taken out from the acid solution, washed with deionized water, and were then dehydrated by tissue paper. The etching time was varied according to the etching rate at different temperatures. Specifically, the optimized etching time conditions were 46 min at − 60 °C, 4 min at 25 °C and 12 s at 80 °C, respectively. Domain patterns were finally studied on a scanning electron microscopy (SEM) JSM-7610F.
